# Supplementary material for: Pyruvate Dehydrogenase Kinase 1 inhibition mediated oxidative phosphorylation enhancement in cartilage promotes osteoarthritis progression
Source: BMC Musculoskelet Disord. 2023 Jul 20;24:597. doi: 10.1186/s12891-023-06585-6 (PMC10357736; doi:10.1186/s12891-023-06585-6)
Supplement: Supplementary file 1 — Additional file 1. [file 12891_2023_6585_MOESM1_ESM.zip › KEGG.Gsea.1653622432464/KEGG_SELENOAMINO_ACID_METABOLISM.html]

Details for gene set KEGG\_SELENOAMINO\_ACID\_METABOLISM[GSEA]

|  || Dataset | 55457\_collapsed\_to\_symbols.cls.cls #OA\_versus\_normal.cls.cls #OA\_versus\_normal\_repos |
| Phenotype | cls.cls#OA\_versus\_normal\_repos |
| Upregulated in class | OA |
| GeneSet | KEGG\_SELENOAMINO\_ACID\_METABOLISM |
| Enrichment Score (ES) | 0.5191732 |
| Normalized Enrichment Score (NES) | 1.7036492 |
| Nominal p-value | 0.009195402 |
| FDR q-value | 0.023717988 |
| FWER p-Value | 0.286 |
Table: GSEA Results Summary

  

Fig 1: Enrichment plot: KEGG\_SELENOAMINO\_ACID\_METABOLISM      
 Profile of the Running ES Score & Positions of GeneSet Members on the Rank Ordered List

  

| SYMBOL | TITLE | RANK IN GENE LIST | RANK METRIC SCORE | RUNNING ES | CORE ENRICHMENT || 1 | BUD23 | BUD23 rRNA methyltransferase and ribosome maturation factor [Source:HGNC Symbol;Acc:HGNC:16405] | 210 | 0.813 | 0.0896 | Yes |
| 2 | PAPSS2 | 3'-phosphoadenosine 5'-phosphosulfate synthase 2 [Source:HGNC Symbol;Acc:HGNC:8604] | 592 | 0.627 | 0.1416 | Yes |
| 3 | PAPSS1 | 3'-phosphoadenosine 5'-phosphosulfate synthase 1 [Source:HGNC Symbol;Acc:HGNC:8603] | 703 | 0.593 | 0.2104 | Yes |
| 4 | MAT2B | methionine adenosyltransferase 2B [Source:HGNC Symbol;Acc:HGNC:6905] | 715 | 0.589 | 0.2863 | Yes |
| 5 | LCMT1 | leucine carboxyl methyltransferase 1 [Source:HGNC Symbol;Acc:HGNC:17557] | 735 | 0.585 | 0.3610 | Yes |
| 6 | HEMK1 | HemK methyltransferase family member 1 [Source:HGNC Symbol;Acc:HGNC:24923] | 742 | 0.583 | 0.4365 | Yes |
| 7 | LCMT2 | leucine carboxyl methyltransferase 2 [Source:HGNC Symbol;Acc:HGNC:17558] | 946 | 0.529 | 0.4897 | Yes |
| 8 | SEPHS1 | selenophosphate synthetase 1 [Source:HGNC Symbol;Acc:HGNC:19685] | 1616 | 0.411 | 0.4912 | Yes |
| 9 | TRMT11 | tRNA methyltransferase 11 homolog [Source:HGNC Symbol;Acc:HGNC:21080] | 1885 | 0.374 | 0.5192 | Yes |
| 10 | AHCYL2 | adenosylhomocysteinase like 2 [Source:HGNC Symbol;Acc:HGNC:22204] | 3099 | 0.240 | 0.4563 | No |
| 11 | GGT1 | gamma-glutamyltransferase 1 [Source:HGNC Symbol;Acc:HGNC:4250] | 5177 | 0.058 | 0.3025 | No |
| 12 | CBS | cystathionine beta-synthase [Source:HGNC Symbol;Acc:HGNC:1550] | 5285 | 0.049 | 0.3006 | No |
| 13 | AHCY | adenosylhomocysteinase [Source:HGNC Symbol;Acc:HGNC:343] | 5307 | 0.047 | 0.3051 | No |
| 14 | AHCYL1 | adenosylhomocysteinase like 1 [Source:HGNC Symbol;Acc:HGNC:344] | 5837 | 0.006 | 0.2648 | No |
| 15 | SEPHS2 | selenophosphate synthetase 2 [Source:HGNC Symbol;Acc:HGNC:19686] | 6303 | -0.027 | 0.2322 | No |
| 16 | MARS1 | methionyl-tRNA synthetase 1 [Source:HGNC Symbol;Acc:HGNC:6898] | 6400 | -0.035 | 0.2293 | No |
| 17 | METTL2B | "methyltransferase 2B, methylcytidine [Source:HGNC Symbol;Acc:HGNC:18272]" | 6522 | -0.044 | 0.2256 | No |
| 18 | SCLY | selenocysteine lyase [Source:HGNC Symbol;Acc:HGNC:18161] | 7044 | -0.083 | 0.1960 | No |
| 19 | MAT1A | methionine adenosyltransferase 1A [Source:HGNC Symbol;Acc:HGNC:6903] | 8753 | -0.213 | 0.0911 | No |
| 20 | GGT5 | gamma-glutamyltransferase 5 [Source:HGNC Symbol;Acc:HGNC:4260] | 9361 | -0.261 | 0.0779 | No |
| 21 | CTH | cystathionine gamma-lyase [Source:HGNC Symbol;Acc:HGNC:2501] | 11108 | -0.444 | 0.0002 | No |
| 22 | MAT2A | methionine adenosyltransferase 2A [Source:HGNC Symbol;Acc:HGNC:6904] | 12852 | -1.066 | 0.0037 | No |
Table: GSEA details [plain text format]

  

Fig 2: KEGG\_SELENOAMINO\_ACID\_METABOLISM      
 Blue-Pink O' Gram in the Space of the Analyzed GeneSet

  

Fig 3: KEGG\_SELENOAMINO\_ACID\_METABOLISM: Random ES distribution      
 Gene set null distribution of ES for **KEGG\_SELENOAMINO\_ACID\_METABOLISM**
